# In vitro Antitumor Properties of Fucoidan-Coated, Doxorubicin-Loaded, Mesoporous Polydopamine Nanoparticles

**DOI:** 10.3390/molecules27238455

**Published:** 2022-12-02

**Authors:** Hongping Xu, Junhong Ling, Han Zhao, Xinyi Xu, Xiao-kun Ouyang, Xiaoyong Song

**Affiliations:** 1 Department of Pharmacy, Zhoushan Hospital of Traditional Chinese Medicine, 355 Xinqiao Road, Zhoushan 316000, China; 2 School of Food and Pharmacy, Zhejiang Ocean University, Zhoushan 316022, China

**Keywords:** mesoporous polydopamine, antitumor, nanomedicine, photothermal synergistic chemotherapy

## Abstract

Chemotherapy is a common method for tumor treatment. However, the non-specific distribution of chemotherapeutic drugs causes the death of normal cells. Nanocarriers, particularly mesoporous carriers, can be modified to achieve targeted and controlled drug release. In this study, mesoporous polydopamine (MPDA) was used as a carrier for the antitumor drug doxorubicin (DOX). To enhance the release efficiency of DOX in the tumor microenvironment, which contains high concentrations of glutathione (GSH), we used N,N-bis(acryloyl)cysteamine as a cross-linking agent to encapsulate the surface of MPDA with fucoidan (FU), producing MPDA-DOX@FU-SS. MPDA-DOX@FU-SS was characterized via transmission electron microscopy, thermogravimetric analysis, and X-ray photoelectron spectroscopy (XPS), and its antitumor efficacy in vitro was investigated. The optimal conditions for the preparation of MPDA were identified as pH 12 and 20 °C, and the optimal MPDA-to-FU ratio was 2:1. The DOX release rate reached 47.77% in an in vitro solution containing 10 mM GSH at pH 5.2. When combined with photothermal therapy, MPDA-DOX@FU-SS significantly inhibited the growth of HCT-116 cells. In conclusion, MPDA-DOX@FU-SS may serve as a novel, highly effective tumor suppressor that can achieve targeted drug release in the tumor microenvironment.

## 1. Introduction

With the continuous development of medicine, various tumor treatment modalities have been gradually developed. Traditional treatment methods include surgery, chemotherapy, and radiotherapy. Chemotherapy is a conventional treatment, but this approach usually causes liver and kidney function damage, cognitive impairment, and other adverse side effects [1]. The efficacy of chemotherapy is reduced by drug resistance [2]. Photothermal therapy is another approach with many advantages, such as high selectivity, minimal invasiveness, and a fast healing rate [3]; however, there are also some disadvantages, such as the uneven distribution of laser heat and limited light penetration. Thus, combining chemotherapeutic treatment with photothermal therapy is a strategy for overcoming the limitations of each individual treatment approach [4,5,6].

Nanoparticle-based drug delivery systems can be used to deliver poorly soluble drugs and reduce the side effects of chemotherapy [7]. Polydopamine (PDA) is an example of a nanocarrier that exhibits good photothermal conversion properties and high biocompatibility. PDA is rich in aromatic rings, amino groups, and catechols, and surface modifications can be implemented to enable drug loading. Mesoporous PDA (MPDA) has a larger specific surface area and higher drug loading capacity (LC) than PDA [8]. Fucoidan (FU), a fucosylsulfate polysaccharide, exhibits antioxidant, anti-inflammatory, antitumor, antibacterial, antiviral, anticoagulant, and antiangiogenic properties [9,10], as well as good biocompatibility in vivo [11,12,13].

Doxorubicin (DOX) is used in tumor treatment [14,15], butexhibits severe nephrotoxicity, which can cause short- and long-term cardiotoxicity, and its clinical applications are limited [16,17]. DOX has been encapsulated in nanocarriers and liposomes to improve its biocompatibility [18,19]. To reduce the side effects of drugs such as DOX, pH- and glutathione (GSH)-responsive drug delivery systems can be used to release loaded drugs in a site-directed manner [20]. The tumor microenvironment is weakly acidic and contains 10 times the GSH concentration of normal cells [7]. Disulfide bonds break in environments characterized by high concentrations of GSH and can therefore be used to establish a drug release mechanism that is responsive to the tumor microenvironment [21]. This extrapolation can be verified using in vitro drug release models. For example, Li et al. examined the drug release behavior of nano-sponges in solutions containing GSH at pH = 5.0 and 7.4 [22].

In this study, to construct an MPDA-based nanomedicine with a GSH-responsive drug release mechanism (MPDA-DOX@FU-SS), we used N,N-bis(acryloyl)cysteamine (BAC) as a disulfide bond-containing cross-linker to encapsulate MPDA with FU [21]. The system exhibited a high release rate in a simulated tumor microenvironment. When combined with photothermal therapy, MPDA-DOX@FU-SS significantly inhibited tumor cell growth, indicating that this novel nanomedicine may serve as a highly effective tumor suppressor.

## 2. Results and Discussion

### 2.1. Fabrication of MPDA

**Figure 1:**
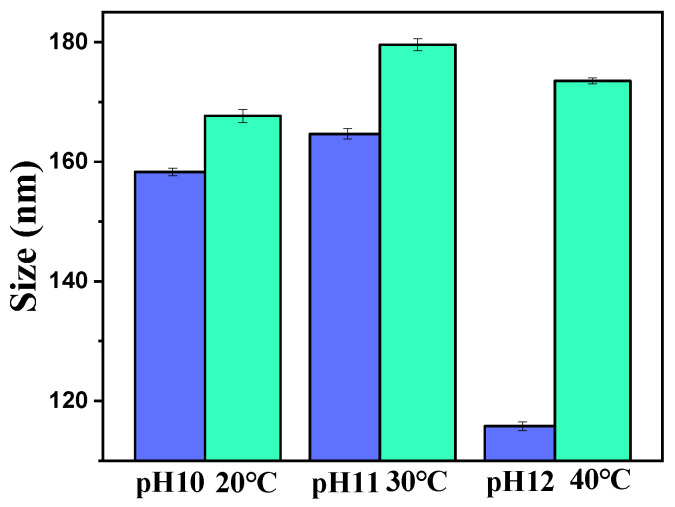
Sizes of mesoporous polydopamine (MPDA) nanoparticles at different pH values and temperatures (Number of samples (n) = 3).

The individual effects of temperature and solution pH on the size of the mesoporous polydopamine (MPDA) nanoparticles were investigated. MPDA was prepared at different temperatures (20, 30, and 40 °C), and the pH of the reaction system was fixed at 9. The resulting particle size was observed (Figure 1). Overall, the effect of temperature on particle size was not significant, and considering the preparation cost, MPDA can be prepared at 20 °C. The effect of pH on particle size is shown in Figure 1. At the same reaction temperature (20 °C), the particle size was smaller at pH 12 compared to that at pH 10 and 11. MPDA was subsequently prepared at pH 12 and 20 °C.

### 2.2. Effect of MPDA to FU Ratio on Particle Size and Surface Potential of MPDA-DOX@FU-SS

**Figure 2:**
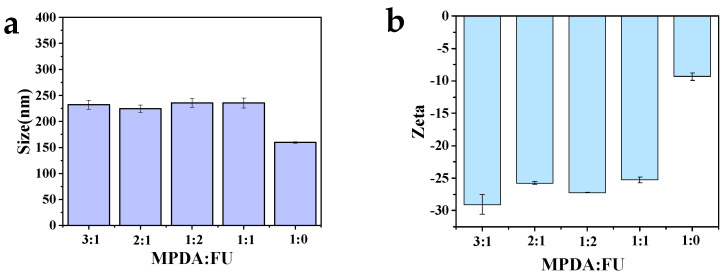
(**a**) Particle size of MPDA−DOX@FU−SS prepared using different ratios of MPDA to fucoidan (FU). (**b**) Zeta potential of MPDA−DOX@FU−SS prepared using different ratios of MPDA:FU (n = 3, DOX: doxorubicin).

The effect of the MPDA to FU ratio on the particle size and surface potential of MPDA-DOX@FU-SS was examined. The size of the MPDA nanoparticles after FU modification increased from 159.7 ± 1.2 nm to 220 nm (Figure 2a). The particle size of MPDA-DOX@FU-SS was smallest (224.7 ± 3.9 nm) when the MPDA to FU ratio was 2:1 (*m/m*), at which point the zeta potential of MPDA-DOX@FU-SS was −25.7 ± 0.2 mV (Figure 2b). As the smallest particle size and best stability were obtained at the MPDA:FU ratio of 2:1, this ratio was used for subsequent experiments.

### 2.3. Scanning Electron Microscopy (SEM) and Transmission Electron Microscopy (TEM) Analysis

**Figure 3:**
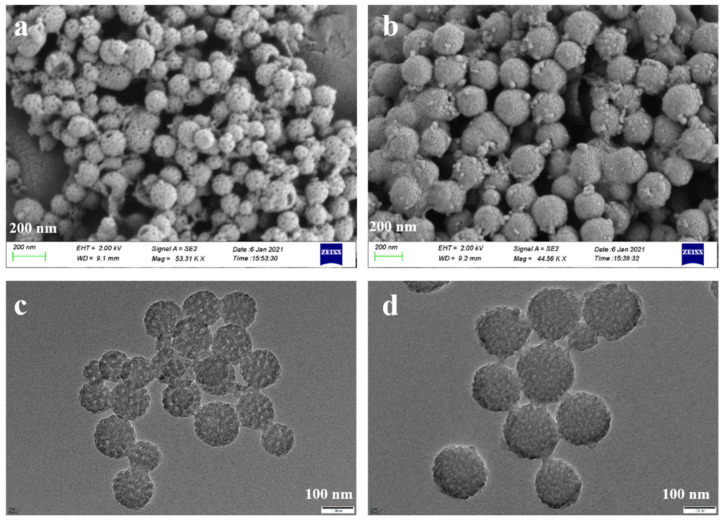
SEM images of (**a**) MPDA and (**b**) MPDA-DOX@FU-SS. TEM images of (**c**) MPDA and (**d**) MPDA-DOX@FU-SS.

The particle size and surface morphology of MPDA and MPDA-DOX@FU-SS were observed using SEM (Figure 3a,b) and TEM (Figure 3c,d). The MPDA nanoparticles exhibited a spherical structure with an average particle size of approximately 120 nm. The structure was uniform and clearly mesoporous. MPDA-DOX@FU-SS had an increased particle size and decreased porosity compared to MPDA, indicating that the nanoparticle surface was successfully coated with FU [23].

### 2.4. Analysis of Fourier Transform Infrared Spectrometer (FTIR) and Analysis of Thermogravimetric Analyzer (TGA)

**Figure 4:**
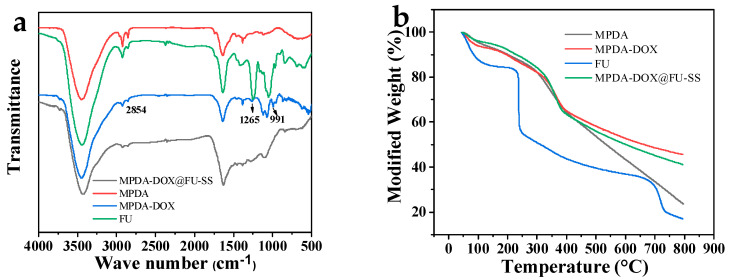
(**a**) Fourier transform infrared spectra and (**b**) thermogravimetric analysis of MPDA, FU, MPDA-DOX, and MPDA-DOX@FU-SS.

The FTIR analysis results for MPDA, FU, MPDA-DOX, and MPDA-DOX@FU-SS are shown in Figure 4a. At 3451 cm^−1^, the absorption peak corresponding to the telescopic vibration process of the phenolic hydroxyl group is visible. The absorption peak at 1550 cm^−1^ corresponds to the bending vibration of the N-H bond and telescopic vibration of the C=C bond. This indicates a favorable outcome of the preparation of PDA [24]. The characteristic absorption peak of the sulfuric acid bond at 846 cm^−1^ is visible in the FTIR spectrogram of FU. The tensile vibration of O=S=O in sulfuric acid shows an absorption peak at 1250 cm^−1^, while the C-O-C stretching vibration of the glycosidic bonds produced an absorption peak at 1024 cm^−1^ [25,26]. In the FTIR spectrum of MPDA-DOX, observable peaks at 2854, 1265, and 988 cm^−1^ were related to quinone and carbonyl groups of DOX [27,28]. After BAC cross-linking, the absorption peak at 2868 cm^−1^ in the FTIR spectrogram of MPDA-DOX@FU-SS, the disappearance of the absorption peaks at 2931 cm^−1^, and the attenuation of the absorption peak at 1250 cm^−1^ are due to the addition of FU coating.

The thermal stabilities of FU, MPDA, and MPDA-DOX@FU-SS were investigated via TGA (Figure 4b). The thermal masses of FU, MPDA, and MPDA-DOX@FU-SS were all less than 20%. At below 100 °C, weight loss of up to 12.53% was mainly attributed to the loss of bound water from FU [29]. At 235–685 °C, the weight loss reached 53.81%, due to the degradation of FU sugar rings and the disassembly of the macromolecular chain. MPDA, MPDA-DOX and MPDA-DOX@FU-SS exhibited similar weight loss in the temperature interval ranging from 37 °C to 400 °C, showing that the addition of DOX and FU does not change the thermal stability of MPDA. This indicates that MPDA-DOX@FU-SS has good thermal stability and can meet the requirements of photothermal chemotherapy for tumor treatment. In the 37–800 °C temperature interval, MPDA-DOX@FU-SS, MPDA, and FU exhibited 59%, 77%, and 84.3% weight loss, respectively. The reduced weight loss of MPDA-DOX@FU-SS indicated that the MPDA surface had been successfully coated with FU, which improved the thermal stability.

### 2.5. LC and Encapsulation Efficiency (EE)

**Figure 5:**
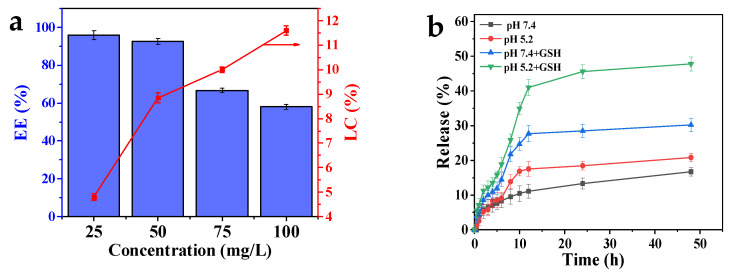
(**a**) DOX loading capacity (blue bar) and encapsulation efficiency (red broken line) of MPDA-DOX@FU-SS. (**b**) Release curves of MPDA-DOX@FU-SS at different pH values and in the presence or absence of glutathione (GSH) (n = 3).

The LC and EE of the carrier were investigated by dispersing 5 mg MPDA in solutions with different concentrations of DOX (25, 50, 75, and 100 mg/L). The optimal carrier-to-DOX concentration ratio was optimized (Figure 5a). The LC increased with the increase in DOX concentration because at increased concentrations, DOX enters the pores of MPDA more easily [30]. When the concentration of DOX was increased from 25 mg/L to 100 mg/L, the EE decreased from 95.96% ± 1.8% to 58% ± 1.2% because the concentration of DOX exceeded the LC of MPDA. Considering the LC and EE, a DOX concentration of 75 mg/L was selected as the optimal concentration for drug loading.

### 2.6. Drug Release Studies In Vitro

Phosphate-buffered saline (PBS; pH 7.40, with or without 10 mM GSH) and acetate buffer solution (ABS; pH 5.2, with or without 10 mM GSH) were used to simulate the tumor cell microenvironment. We investigated the in vitro release behavior of MPDA-DOX@FU-SS using dialysis (Figure 5b). The in vitro release of DOX from MPDA-DOX@FU-SS was pH dependent. With a decrease in the pH, the release rate and released amount of DOX increased. At pH 7.4, the total release rate of MPDA-DOX@FU-SS was 16.71% ± 1.22%, and at pH 5.2, the release rate was 20.82% ± 1.21%. This increase in the release rate occurred because the FU coating and PDA nanoparticles were destroyed in the low-pH environment [31]. The MPDA-DOX@FU-SS release rate in the solution containing GSH was significantly higher than that in the solution lacking GSH, indicating GSH-dependent release. At pH 5.2, in the presence of GSH, the cumulative MPDA-DOX@FU-SS release rate was 47.77% ± 2.05%, while in the absence of GSH, the release rate was only 20.82% ± 1.21%. At pH 7.4, in the presence of GSH, the MPDA-DOX@FU-SS cumulative release rate was 30.21% ± 1.95%, indicating that GSH dependence had a stronger effect than pH dependence. When GSH is present, the disulfide bond in the nanodrug breaks, promoting DOX release. The results confirmed that MPDA-DOX@FU-SS contained a disulfide bond.

### 2.7. XPS Analysis

**Figure 6:**
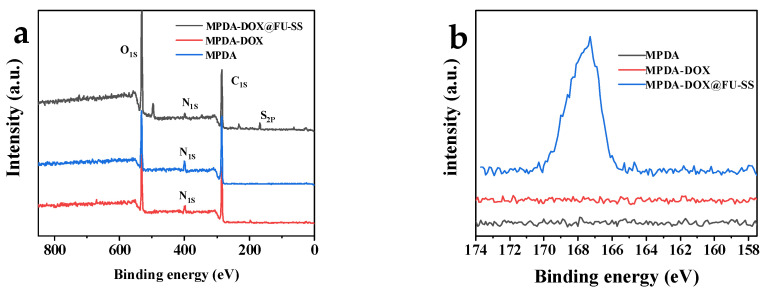
X-ray photoelectron spectroscopy spectrum. (**a**) Full spectrum. (**b**) X-ray photoelectron spectroscopy comparison diagram of S.

XPS was performed for element composition analysis of the carrier before and after drug loading and before and after the FU coating (Figure 6). As shown in Figure 6a,b, the main elements in MPDA were C, N, and O. The content of N in MPDA increased after DOX loading. In addition, a characteristic S peak was observed, which mainly originated from the disulfide bond that formed after FU was cross-linked via BAC. The presence of the S element peak indicates that FU was successfully coated onto the surface of MPDA.

### 2.8. Photothermal Effect and N_2_ Adsorption and Desorption Analysis

**Figure 7:**
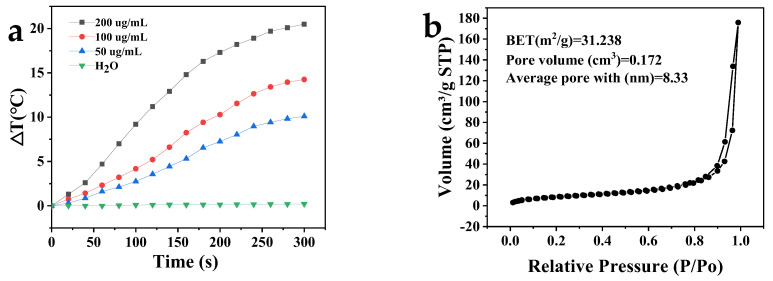
(**a**) Temperature variation of nanoparticle solutions containing different concentrations of MPDA. (**b**) N_2_ adsorption and desorption curves of MPDA. BET, Brunauer–Emmett–Teller.

The photothermal conversion performance of the MPDA nanoparticles is shown in Figure 7a. At different concentrations of MPDA (200, 100, and 50 μg/mL), the temperature in aqueous MPDA dispersions increased by 20.5 °C, 14.3 °C, and 10.1 °C after near infrared (NIR) irradiation (808 nm, 2 W/cm^3^) for 5 min. There was no change in the temperature of the deionized water control solution after NIR radiation, indicating that the increased temperature of the aqueous MPDA dispersions occurred because of the photothermal conversion effect of MPDA [32]. When the MPDA concentration was 100 μg/mL, the solution temperature reached 42.3 °C after NIR (808 nm, 2 W/cm^3^) irradiation for 5 min. The lethal temperature of most tumor cells is 42–43 °C, indicating that MPDA can be applied as a photothermal antitumor agent.

The specific surface area of MPDA was determined to be 31.238 m^2^/g via the Brunauer–Emmett–Teller (BET) method (Figure 7b). The nitrogen adsorption and desorption curves of MPDA conformed to those of type IV isotherms, indicating that MPDA was a mesoporous material [33]. This finding was consistent with the SEM and TEM characterization results. The MPDA mesoporous volume and diameter, analyzed using the Barrett–Joyner–Halenda method, were 0.171904 cm^3^/g and 8.335 nm, respectively. The above results indicate that MPDA had a high specific surface area and pore volume and that it could be used to deliver drug payloads.

### 2.9. Cytotoxicity

**Figure 8:**
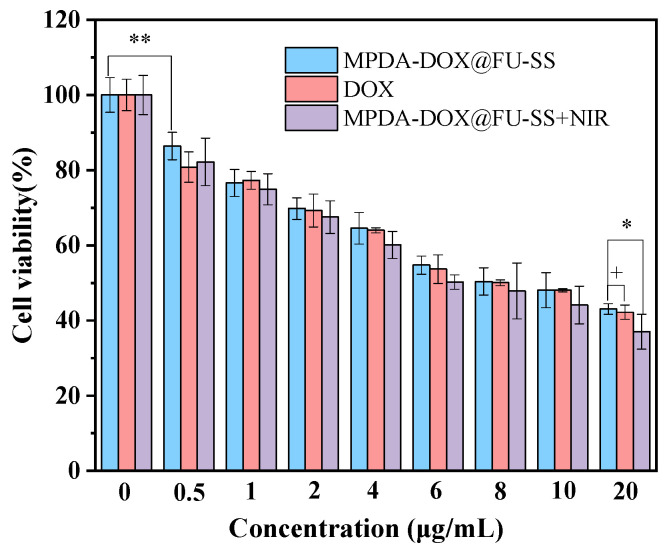
HCT-116 cell viability rate after 24 h of treatment with MPDA-DOX@FU-SS, DOX, and MPDA-DOX@FU-SS+NIR (n = 3; ** *p* < 0.01, * 0.05 > *p* > 0.01, ^+^ *p* > 0.05).

The in vitro cytotoxicity of DOX, MPDA-DOX@FU-SS, and MPDA-DOX@FU-SS+NIR in HCT-116 tumor cells was analyzed using the 3-(4,5-dimethylthiazol-2)-2,5-diphenyltetrazolium bromide (MTT) assay (Figure 8). The HCT-116 cell survival rate gradually decreased with increasing drug concentrations. HCT-116 tumor cells were handled with DOX and MPDA-DOX@FU-SS (5 μg/mL to 20 μg/mL). After 24 h, the viability of the HCT-116 tumor cells gradually decreased in a dose-dependent manner. As shown in Figure 8, the survival rate of HCT-116 tumor cells was 42.21 ± 1.89% after 24 h of culture with 20 μg/mL DOX and 37.01 ± 4.613% after 24 h of culture with MPDA-DOX@FU-SS+NIR. MPDA-DOX@FU-SS+NIR exhibited the best cytotoxicity at the same dosage. In conclusion, MPDA-DOX@FU-SS had good tumor cytotoxicity when combined with NIR light irradiation.

### 2.10. Fluorescent Staining of HCT-116 Cells

**Figure 9:**
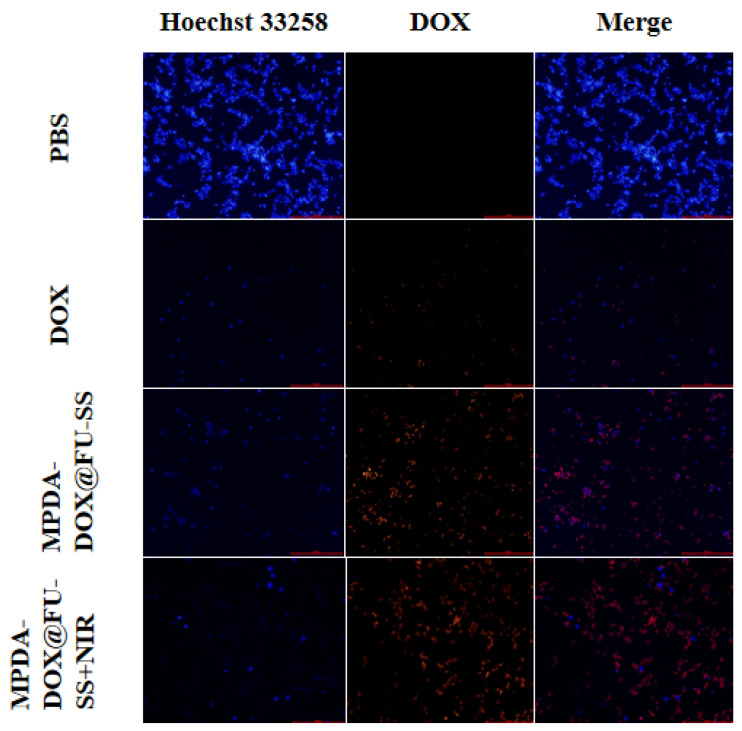
Fluorescence diagrams of cells treated with phosphate-buffered saline (PBS), free DOX, MPDA-DOX@FU-SS, or MPDA-DOX@FU-SS + near infrared (NIR) for 12 h.

HCT-116 tumor cells were treated with PBS, DOX, MPDA-DOX@FU-SS, or MPDA-DOX@FU-SS+NIR for 12 h. The cellular uptake of MPDA-DOX@FU-SS was observed using inverted fluorescence microscopy (Figure 9). After 12 h of incubation, the red fluorescence intensity of HCT-116 tumor cells in the DOX culture group was lower than that in the MPDA-DOX@FU-SS+NIR culture group because DOX entered the cells via free diffusion or cytokinesis. However, DOX was poorly retained in tumor cells, whereas MPDA-DOX@FU-SS, with enhanced permeability and retention effects, was extensively retained in tumor cells. DOX was released under NIR irradiation in a microenvironment characterized by a low pH and high GSH concentration [34,35]. The MPDA-DOX@FU-SS+NIR delivery system exhibited improved drug uptake by tumor cells.

## 3. Materials and Methods

### 3.1. Materials

DOX hydrochloride (DOX·HCl) was obtained from Haoyun Chemical Technology Co., Ltd. (Shanghai, China). FU, ethyl orthosilicate, potassium bromide, N-hydroxysuccinimide (NHS), 1-(3-dimethylaminopropyl)-3-ethylcarbodiimide hydrochloride (EDC·HCl), dopamine hydrochloride (DA·HCl), 1,3,5-trimethylbenzene (TMB), ammonia (NH_3_·H_2_O), and Pluronic F127 (F127) were obtained from Aladdin Reagent Co., Ltd. (Shanghai, China). BAC was obtained from Sigma-Aldrich Trading Co., Ltd. (Shanghai, China).

### 3.2. Methods

#### 3.2.1. MPDA Preparation

MPDA was prepared via the one-pot method as described previously, with some adjustments [36,37]. First, 250 mg F127 and 100 μL TMB were added to a mixture of 7.5 mL ethanol and 7.5 mL water and dissolved by ultrasonic shaking to obtain a mixture of F127 and TMB. After 75 mg of DA·HCl was added to the mixture, the pH was adjusted by adding NH_3_·H_2_O drop wise and by continuous stirring of the reaction mixture at 20 °C for 24 h. The precipitate was added to 40 mL of acetone and ethanol (1:2, *v*/*v*) and washed three times by ultrasonic shaking for 30 min to remove the F127. Finally, the products MPDA-1, MPDA-2, and MPDA-3 were obtained. This procedure was repeated, and MPDA was prepared at 20, 30, and 40 °C.

#### 3.2.2. MPDA-DOX@FU-SS Preparation

**Figure 10:**
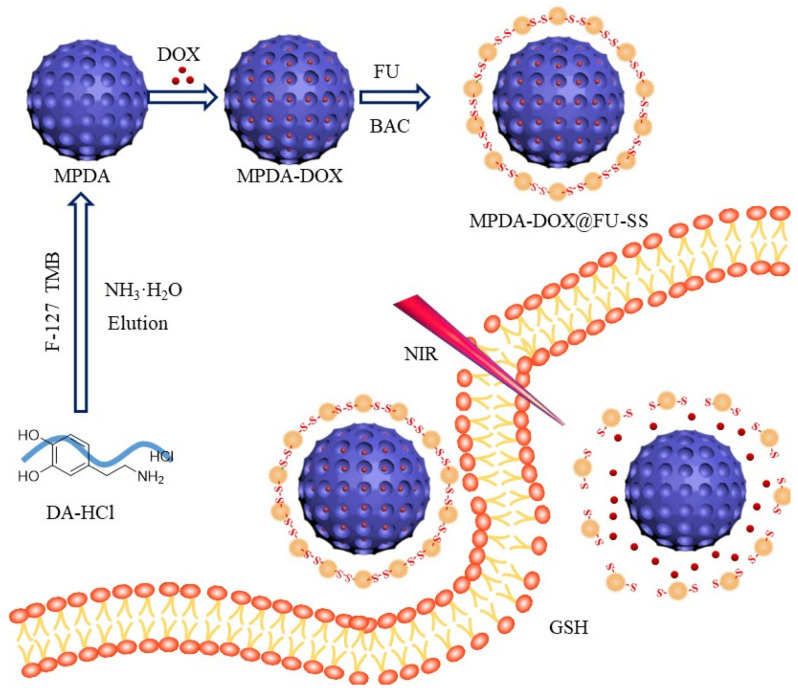
Schematic diagram of the preparation and release of MPDA-DOX@FU-SS nanoparticles.

A schematic of the preparation of the MPDA-DOX@FU-SS nanodrug is shown in Figure 10. DOX (25 mg) was dissolved in 50 mL of deionized water by ultrasonication under light-proof conditions. A 250 mL volumetric flask was used to prepare a 100 mg/L DOX stock solution with a fixed volume. MPDA (5 mg) was added to 10 mL of the DOX solution and stirred at room temperature for 24 h. FU (50 mg) was added to PBS (50 mL) containing 100 mg of NHS and 100 mg of EDC·HCl, and the solution was stirred for 2 h at room temperature. Next, 2 mL of ethylenediamine was added drop by drop to obtain the activated FU solution. After the reaction, the sediment was washed with water centrifugation and ethanol (12,000 rpm, 10 min) to remove residual FU and MPDA-DOX@FU-SS.

#### 3.2.3. Material Characterization

The functional groups of the resulting nanomaterials were analyzed using Fourier transform infrared spectrometer (Tensor II, Bruker Corp., Karlsruhe, Germany) in the wavenumber range of 4000–400 cm^−1^. SEM (S-4800, Hitachi Corp., Tokyo, Japan) was used to evaluate the surface morphology of the nanoparticles. All nanoparticles were uniformly blown onto the silicon wafer. The samples were vacuum coated with a thin gold film before analysis and then observed at an accelerating voltage of 20 kV at an appropriate magnification. TEM (JEM-2100, Hitachi Corp., Tokyo, Japan) analysis of the samples was performed using a Lorentz transmission electron microscope. An appropriate amount of nanoparticle solution was evenly dropped onto the copper network and observed. The surface zeta potential and the particle size were measured using a zeta potentiometer (Nano ZS, Malvern, PA, USA). The specific surface area, pore volume, and pore size of MPDA were determined using the BET method (Tri Star II 3020, Micromeritics, Atlanta, GA, USA). XPS (ESCALAB, Thermo Fisher Scientific, Waltham, MA, USA) was used to analyze the elemental changes in the products at each stage of the preparation process. The obtained nanoparticles were subjected to TGA (TGA55, TA Instruments, Newcastle, Delaware, USA) using a Q50 thermal analyzer with a temperature rise rate of 10 °C/min in an N_2_ atmosphere of 37–800 °C. Various amounts of MPDA nanoparticles were dispersed in deionized water, and 150 μL of each MPDA dispersion (0, 50, 100, and 200 μg/mL) was configured and irradiated with NIR light (808 nm, 2 W/cm^3^) for 300 s. The temperature changes were recorded every 20 s to investigate the photothermal conversion performance of MPDA.

#### 3.2.4. LC and EE

The DOX concentration was determined using the UV spectrophotometric method. DOX (10 mg) was accurately weighed, and a 10 mg/mL DOX master batch was prepared. The mother liquor was diluted to obtain DOX standard working solutions of 1.6, 4, 8, 10, and 16 μg/mL, and absorbance values were measured at 480 nm to plot the DOX standard curve. The MPDA-DOX solution was centrifuged at 12,000 rpm for 10 min, and the supernatant was collected. The initial mass of DOX was recorded as *W*_0_. *W_e_* denotes the multiples of free DOX in the supernatant, and *W* is the sample mass. The EE and LC of DOX were calculated using the following equations:

(1) $$\mathrm{EE}=\frac{W_{e}}{W_{0}}\times 100\%$$

(2) $$\mathrm{LC}=\frac{W_{0}-W_{e}}{W}\times 100\%$$

where *W_e_* is the amount of DOX loaded onto MPDA and *W*_0_ is the amount of DOX added during the preparation procedure. *W* is the weight of the MPDA.

#### 3.2.5. Drug Release In Vitro

DOX (5 mg) was weighed and dispersed in 10 mL of PBS (pH 7.4) or ABS (pH 5.2) with/without GSH (10 mM). The solution was transferred to a dialysis bag (MWCO: 3000 KDa) and placed in 200 mL of the same buffer at 37 °C. The solution was shaken (150 rpm) at a constant temperature, and the supernatant (3 mL) was removed by centrifugation at 0.5, 1, 2, 3, 4, 5, 6, 8, 10, 12, and 24 h (equal amounts of buffer were added after sampling). The amount of DOX in the dialysate was measured.

#### 3.2.6. Nanoparticle Cytotoxicity Assessment

The cytotoxicity of the MPDA-DOX@FU-SS nanodrug was assessed by determining the survival rate of HCT-116 tumor cells. Cell viability and growth rate were determined using the MTT assay [38,39]. HCT-116 cells were cultured in DMEM (10% fetal bovine serum, 100 μg/mL streptomycin, and 100 μg/mL penicillin) until the cell density in the culture flasks was 80–90%; 180 μL of medium per well (50,000 cells) was added to 96-well plates and incubated at 37 °C under 5% CO_2_ and saturated humidity for 24 h. Culture medium (20 µL/well) containing different concentrations of DOX or MPDA-DOX@FU-SS (0.5, 1, 2, 4, 6, 8, 10, 20 µg/mL) was added for 24 h. Next, 20 µL of MTT solution (5 mg/mL) was added to each well under light-proof conditions. After 4 h of incubation, 150 μL of dimethyl sulfoxide was added to each well to replace the culture medium, and the absorbance of the solution in each well was measured using a microplate reader at 490 nm (Infinite 200 PRO, Tecan, Männedorf, Switzerland) after shaking for 15 min [40]. The absorbance of the wells containing the drug was recorded as *OD*_1_, and the absorbance of the control wells was recorded as *OD*_0_. Tumor cell viability was calculated using the following equation

(3) $$Viability\;\left(\%\right)=\frac{OD_{1}}{OD_{0}}\times 100\%$$

#### 3.2.7. Nanocarrier Cellular Uptake Experiment

The nanocarrier uptake efficiency was explored in HCT-116 tumor cells. Nanocarrier-loaded DOX emitted red fluorescence under green laser irradiation, which was observed via fluorescence microscopy [41]. HCT-116 cells were inoculated into 6-well culture plates (2 mL of medium per well) at a cell density of 1.5 × 10^6^/well and cultured for 24 h. The DOX concentration was set at 10 μg/mL per group, and 1 mL of DOX solution, PBS, MPDA-DOX@FU-SS, or MPDA-DOX@FU-SS (containing 10 mM GSH) were added to the 6-well plates. The plates were incubated at 37 °C under 5% CO_2_ and saturated humidity for 12 h. The culture solution was removed and washed three times with PBS, and 1 mL of Hoechst 33,258 stain (10 μg/mL) was added to the solution, which was then incubated for 30 min. Next, the stain was discarded. The solution was washed with PBS and placed in an inverted microscope for fluorescence intensity analysis [42].

## 4. Conclusions

MPDA-DOX@FU-SS is a synergistic photothermal/chemotherapeutic nano-therapy platform. The optimal conditions for the preparation of MPDA were pH 12 and 20 °C. The optimal MPDA-to-FU ratio was 2:1. XPS showed that MPDA and FU could be bound by a disulfide bond. The DOX release rate reached 47.77% in an acidic environment with a high GSH concentration, indicating that this system can achieve targeted release in the tumor microenvironment. The MTT assay revealed that MPDA-DOX@FU-SS significantly inhibited the growth of HCT-116 cells. The results of cell uptake showed that MPDA-DOX@FU-SS can significantly increase the uptake of DOX by tumor cells. Therefore, MPDA-DOX@FU-SS may serve as a highly effective tumor suppressor because of its synergistic effect of photothermal therapy and chemotherapy.

## Data Availability

Not applicable.

## Sample Availability

Samples of the compounds are not available from the authors.
